# Supplementary material for: Evaluating Targeted Therapies in Ovarian Cancer Metabolism: Novel Role for PCSK9 and Second Generation mTOR Inhibitors
Source: Cancers (Basel). 2021 Jul 24;13(15):3727. doi: 10.3390/cancers13153727 (PMC8345177; doi:10.3390/cancers13153727)
Supplement: Supplementary file 1 [file cancers-13-03727-s001.zip › cancers-1228664-supplementary.pdf]

Supplementary Materials: Evaluating Targeted Therapies in Ovarian Cancer Metabolism: Novel Role for PCSK9 and Second Generation mTOR Inhibitors

Dafne Jacome Sanz, Juuli Raivola, Hanna Karvonen, Mariliina Arjama, Harlan Barker, Astrid Murumägi and Daniela Ungureanu

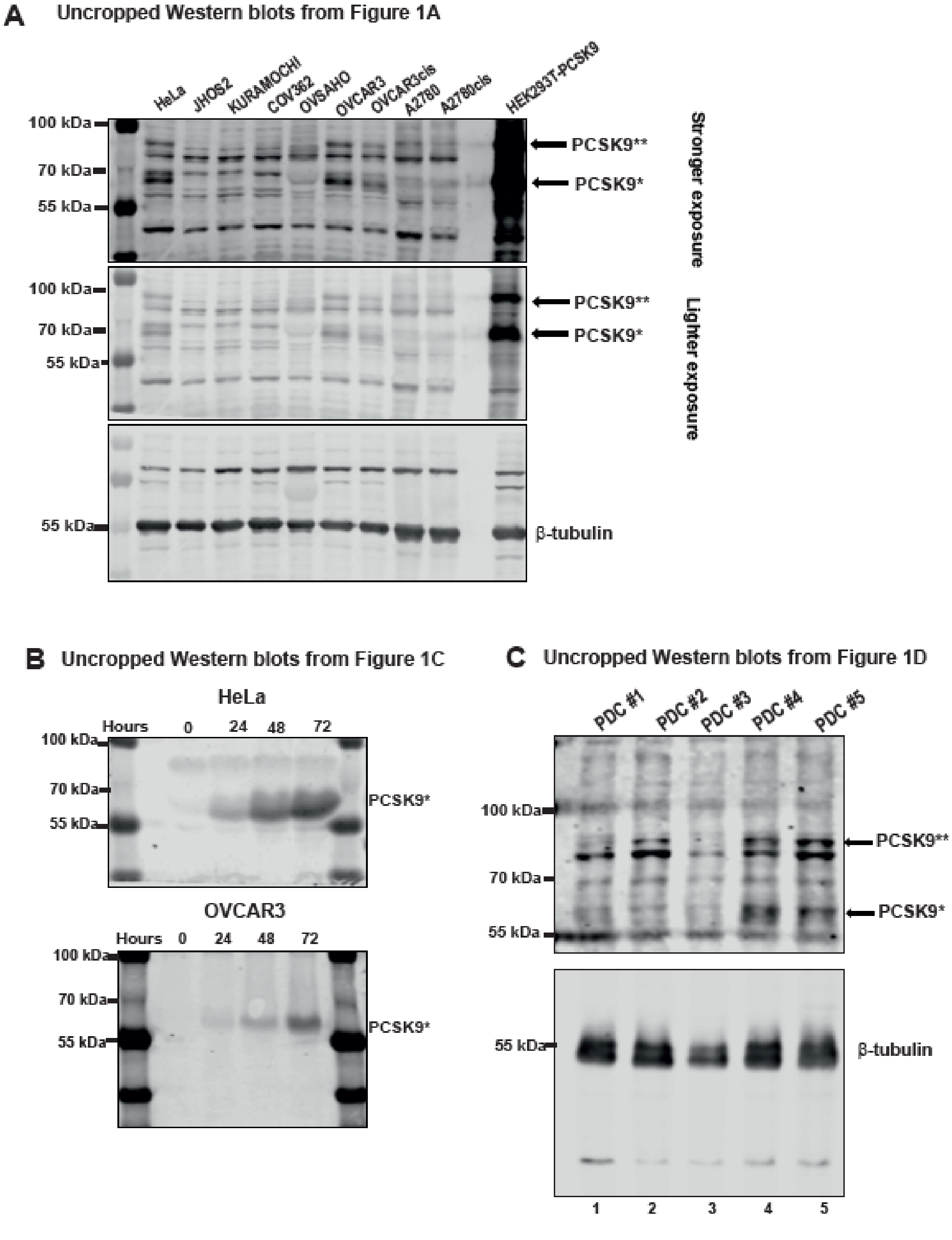

Figure S1. Analysis of PCSK9 isoforms in OC models. (**A**) Upper panel: Uncropped Western blot of Figure 1A showing PCSK9 expression in the OC cell lines. For an accurate analysis, a sample of over-expressed PCSK9 in HEK293T cells is shown as HEK293T-PCSK9. Lower panel: β-tubulin levels as loading control. (**B**) Uncropped Western blots from Figure 1C. (**C**) Uncropped Western blots from Figure 1D.

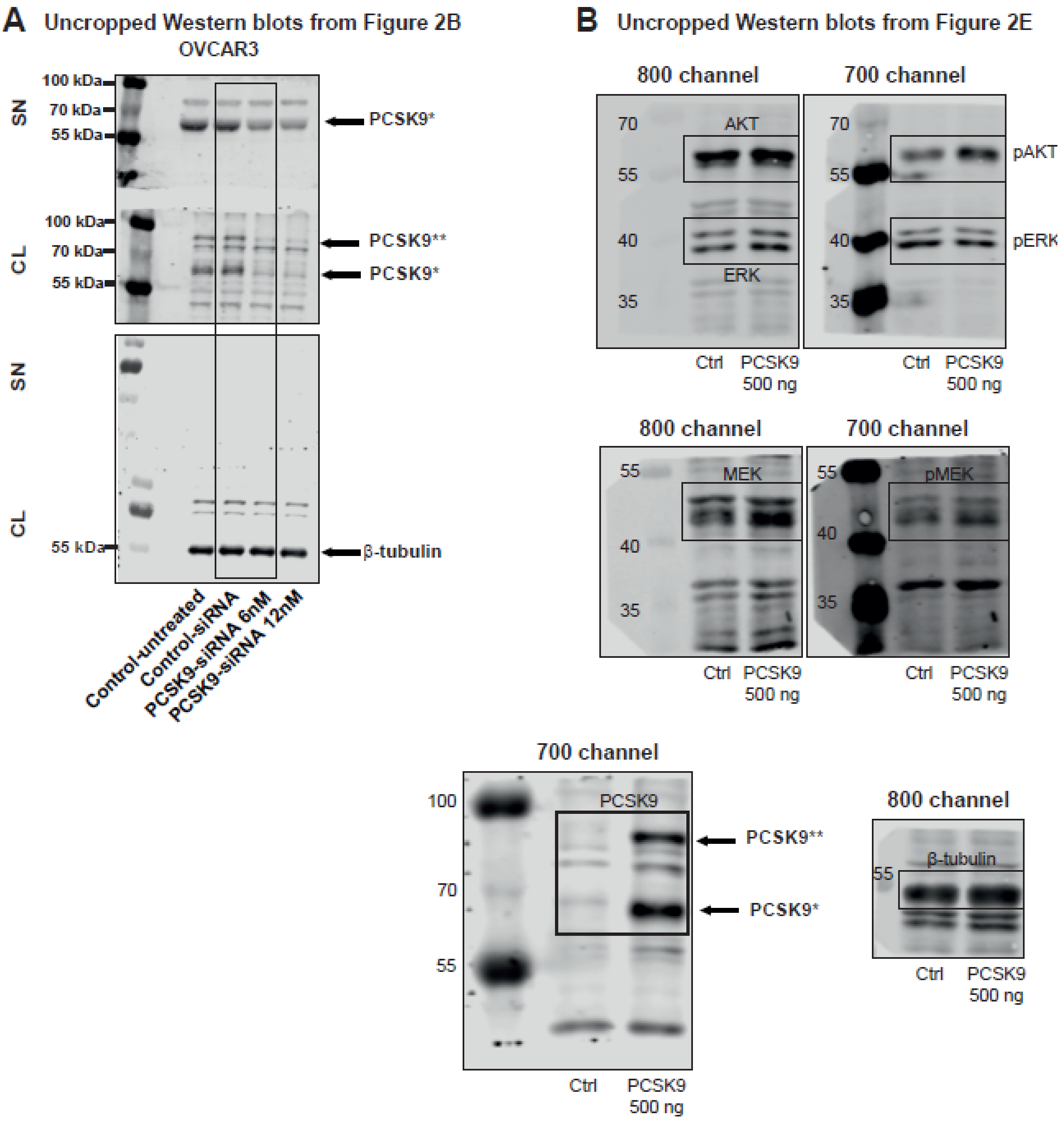

Figure S2. Analysis of PCSK9 targeting in OC cell line. (**A**) Upper panel: Uncropped Western blot of Figure 2B showing PCSK9 expression in OVCAR3 cells treated with Control-siRNA or PCSK9-siRNA for 48h, for supernatant (SN) and cell lysate fractions (CL). Lower panel: β-tubulin levels as loading control. (**B**) Uncropped Western blots for Figure 2E showing the expression of indicated protein from JHOS2 ctrl or PCSK9 (500 ng) transfection. Frames in blots indicate the bands shown in Figure 2E. Ladder molecular weights in kDas.

(**A**)

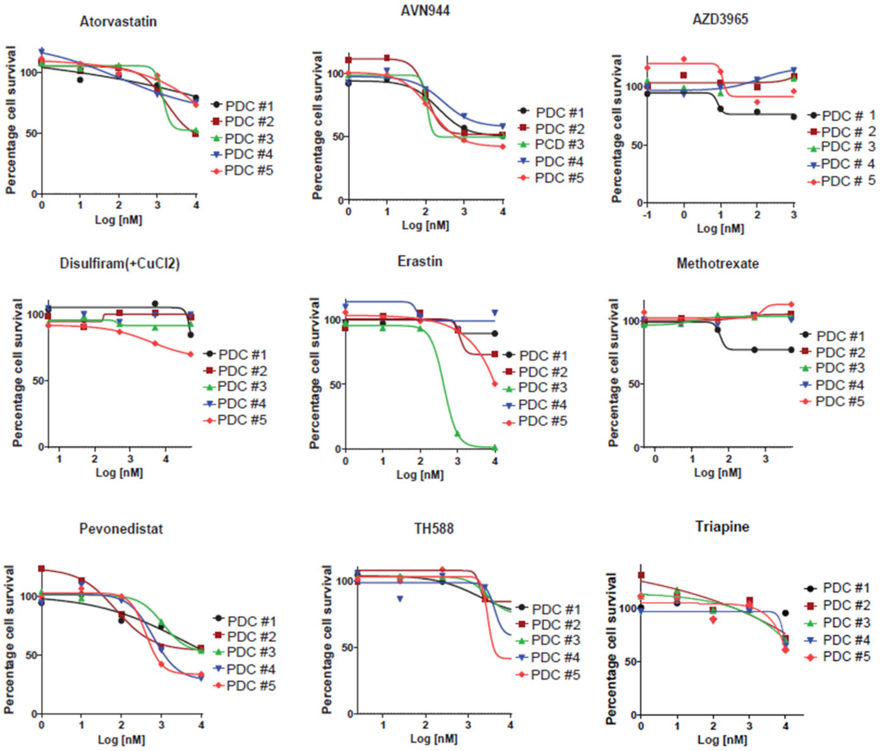

(**B**)

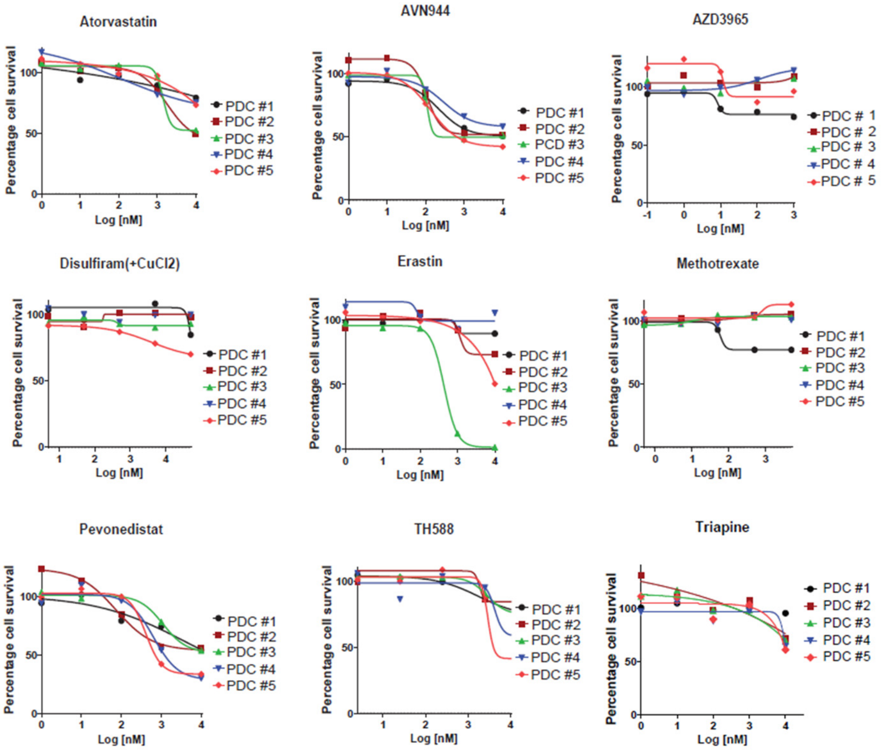

Figure S3. Original drug-response curves of anti-metabolic drugs (n = 11) in OC cell lines (**A**) and PDCs (**B**). For each drug, five concentrations are represented as log10 values, and cell viability is calculated as percentage inhibition, where the lowest drug concentration is given 100% percentage cell survival.

(**A**)

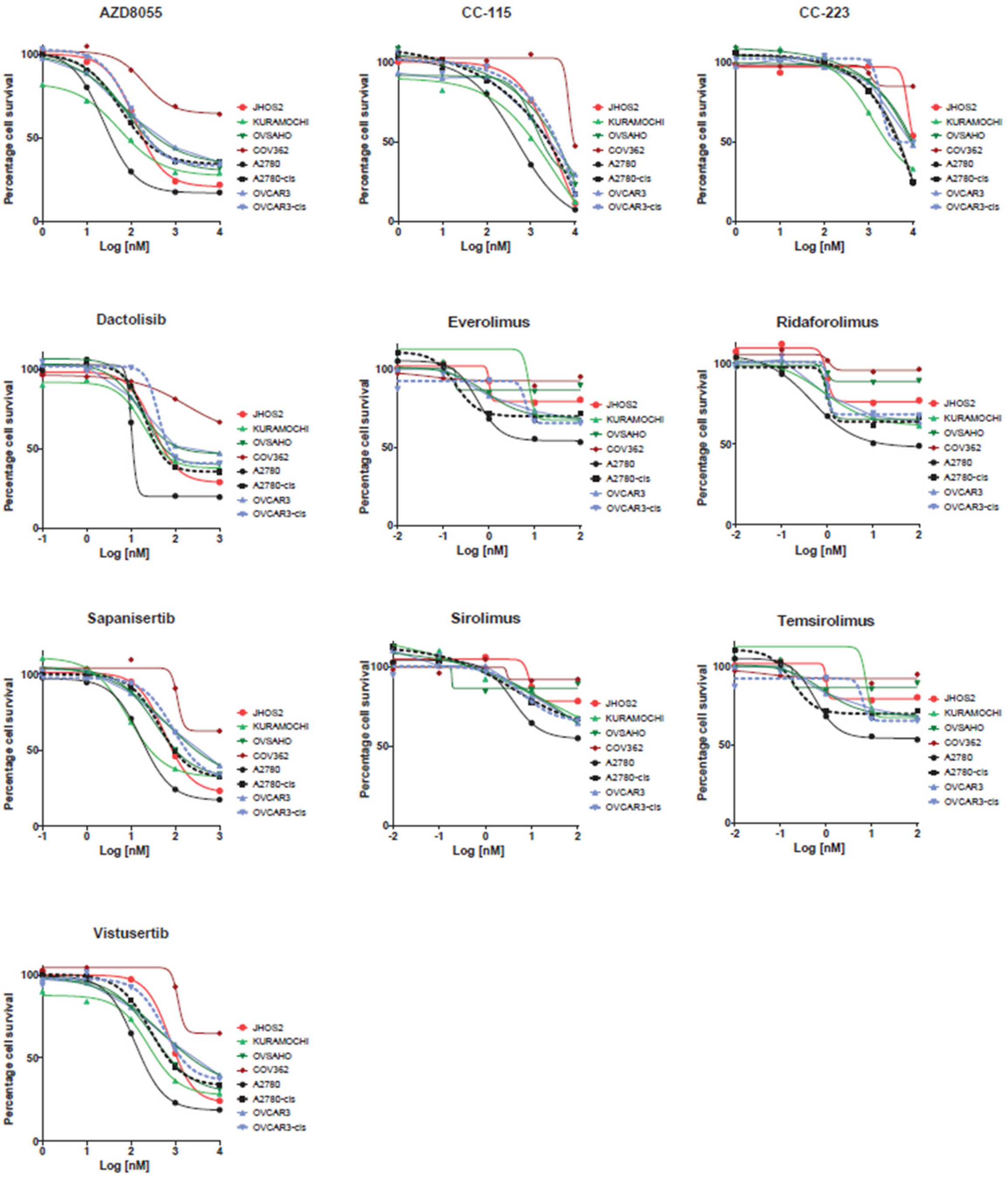
AZD8055
CC-115
CC-223
Dactolisib
Everolimus
Ridaforolimus
Sapanisertib
Sirolimus
Temsirolimus
Vistusertib
Percentage cell survival
Log [nM]
JHOS2
KURAMOCHI
OVSAHO
COV362
A2780
A2780-cis
OVCAR3
OVCAR3-cis

(**B**)

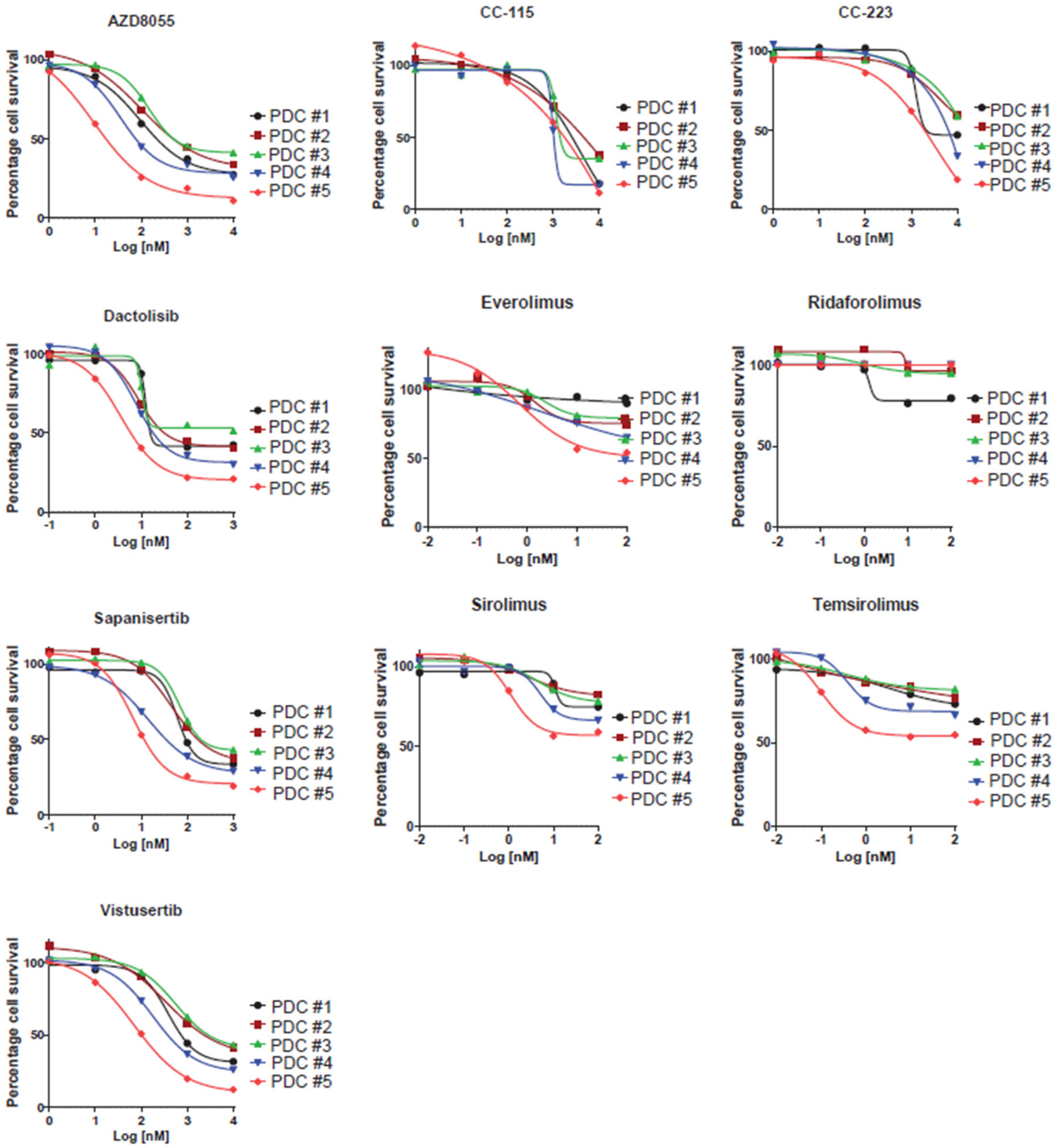

Figure S4. Original drug-response curves of mTOR targeting drugs (n = 10) in OC cell lines (**A**) and PDCs (**B**). For each drug, five concentrations are represented as log M values, and cell viability is calculated as percentage inhibition, where the lowest drug concentration is given 100% percentage cell survival.

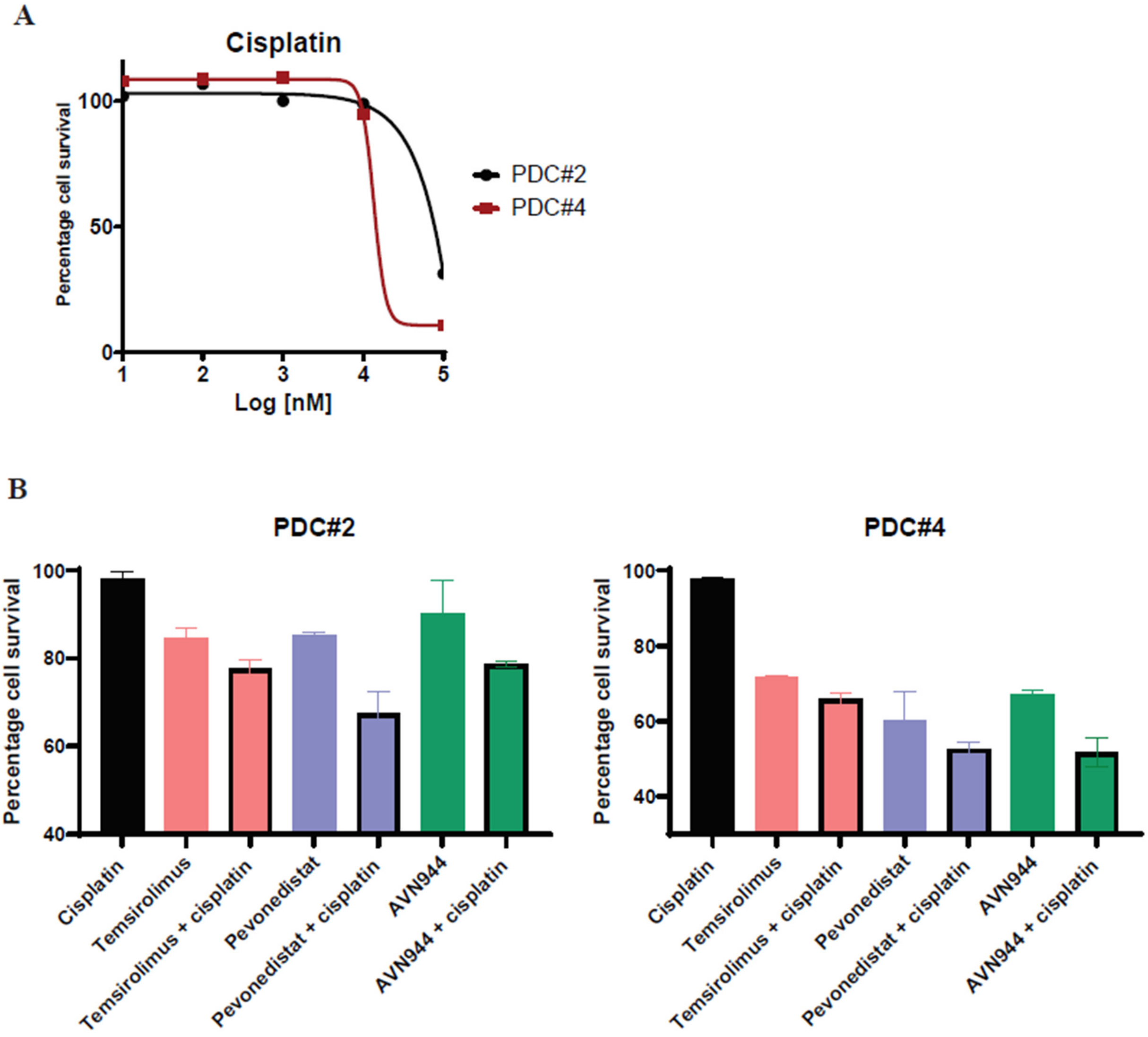

Figure S5. The effect of cisplatin (**A**) and cisplatin in combination with other drugs (**B**) on PDC#2 and PDC#4. (**A**) Drug response curves for cisplatin sensitivity in PDC#2 and PDC#4. Five concentrations of cisplatin are represented as log M values and cell viability is calculated as percentage inhibition, where the lowest cisplatin concentration is given 100% percentage cell survival. (**B**) Drug combination using cisplatin together with either temsirolimus, pevonedistat and AVN944 showed enhanced cytotoxic activity in both, PDC#2 and PDC#4 when both drugs were added, compared to either treatment alone. For drug combinations, a concentration of 1 µM cisplatin was chosen, as it had no effect on cell survival in (A). Other drugs were used at concentration of 1 nM (temsirolimus) and 100 nM (pevonedistat and AVN944). PDCs were treated with drugs or drug-combinations as indicated for 72 h and cell viability was calculated as percentage inhibition compared to vehicle (DMSO) control (100%). Each experiment was performed in duplicates and error bars indicate standard deviations.

Table S1. Signal values for of the Western blots in Figure 1.

	**Figure 1A**				
PCSK9**	Channel	Name	Signal	Signal	Signal
	700	HeLa	174	111	1540
	700	JHOS2	32,9	0,291	219
	700	Kuramochi	38,2	23,9	440
	700	COV362	57,6	35,9	729
	700	OVSAHO	66	6,13	152
	700	OVCAR3	213	216	1660
	700	OVCAR3cis	336	208	623
	700	A2780	41,3	35	716
	700	A2780cis	44,9	28,5	548
PCSK9*	Channel	Name	Signal	Signal	Signal
	700	HeLa	416	271	2720
	700	JHOS2	57,6	28,5	642
	700	Kuramochi	13,6	29	874
	700	COV362	203	84,7	1330
	700	OVSAHO	59,8	19	182
	700	OVCAR3	444	463	1280
	700	OVCAR3cis	951	652	716
	700	A2780	5,95	51,9	261
	700	A2780cis	11,1	67,9	428
Tubulin	Channel	Name	Signal	Signal	Signal
	800	HeLa	711	1390	5280
	800	JHOS2	583	1000	4200
	800	Kuramochi	616	1560	4280
	800	COV362	1190	2360	5220
	800	OVSAHO	372	749	3340
	800	OVCAR3	698	1550	3030
	800	OVCAR3cis	803	1610	3250
	800	A2780	647	1570	4610
	800	A2780cis	686	1010	4110
	**Figure 1C**				
		**OVCAR3**			
**PCSK9****	Channel	Name	Signal		
	700	0	4510		
	700	24	13200		
	700	48	47400		
	700	72	166000		
		HeLA			
**PCSK9***	Channel	Name	Signal		
	700	0	158		
	700	24	2510		
	700	48	13700		
	700	72	26300		
	**Figure 1E**				
**PCSK9****	**Channel**	**Name**	Signal	Signal	Signal
	700	PDC #1	16,9	19,9	29,1
	700	PDC #2	20,6	41,9	49,9
	700	PDC #3	12	9,87	9,1
	700	PDC #4	32	66,6	65,3
	700	PDC #5	52,7	76,9	77
**PCSK9***	Channel	Name	Signal	Signal	Signal
	700	PDC #1	32,3	43,9	36,2
	700	PDC #2	30,3	26,9	26,6
	700	PDC #3	16,1	26	17,3
	700	PDC #4	80,2	120	94,3
	700	PDC #5	149	108	100
**Tubulin**	Channel	Name	Signal	Signal	Signal
	800	PDC #1	11300	11300	11300
	800	PDC #2	11600	11600	11600
	800	PDC #3	8170	8170	8170
	800	PDC #4	11700	11700	11700
	800	PDC #5	11900	11900	11900

Table S2. Signal values from the western blots in Figure 2.

Signals for quantifications in 2C							
**Channel**	**Antibody/band**	**OVCAR3 – Ctr-siRNA**			**OVCAR3 – PCSK9-siRNA**		
700	PCSK9* SN	1990	1910	4160	670	466	1390
700	PCSK9** CL	433	114	1390	75.7	14.8	605
700	PCSK9* CL	928	525	3170	144	72	947
800	β-tubulin	14500	1020	5340	12800	951	6120

Signals for quantifications in 2F							
**Channel**	**Antibody**	**JHOS2 - Ctrl**			**JHOS2 - PCSK9**		
800	AKT	2630	3380	2530	2910	3550	2870
700	pAKT (Ser 473)	611	734	548	958	1030	855
800	MEK1/2	813	741	1320	1020	642	1650
700	pMEK1/2 (Ser217/221)	256	415	407	304	344	532
800	ERK1/2	1430	2230	1070	1920	2620	1470
700	pERK1/2 (Thr202/Tyr204)	1000	3270	1730	811	4010	2220
800	β-tubulin	671	4570	1620	713	3890	1700
